# Systematic Review and Meta-analysis: The Association Between Child and Adolescent Depression and Later Educational Attainment

**DOI:** 10.1016/j.jaac.2020.10.008

**Published:** 2021-01

**Authors:** Alice Wickersham, Holly V.R. Sugg, Sophie Epstein, Robert Stewart, Tamsin Ford, Johnny Downs

**Affiliations:** a Institute of Psychiatry, Psychology and Neuroscience, King’s College London, United Kingdom; b South London and Maudsley NHS Foundation Trust, London, United Kingdom; c University of Exeter Medical School, Exeter, United Kingdom; d University of Cambridge, United Kingdom

**Keywords:** depression, educational status, meta-analysis

## Abstract

**Objective:**

The association between depression and educational attainment in young people is unclear. This systematic review and meta-analysis examines the longitudinal association between depression and subsequent attainment, and its potential effect modifiers and mediators.

**Method:**

We searched Embase, PsycINFO, PubMed, ERIC, and the British Education Index from inception to October 23, 2019, conducted citation searching, and contacted authors for articles. Eligible studies reported on the longitudinal association between depression in children and adolescents 4 to 18 years of age and later educational attainment. Two reviewers independently conducted screening, data extraction, and risk of bias assessment. Correlation coefficients were pooled in meta-analysis, and effect modifiers were explored using meta-regression and stratification. Other evidence on confounders, modifiers, and mediators was narratively synthesized. The PROSPERO record for the study is CRD42019123068.

**Results:**

A total of 31 studies were included, of which 22 were pooled in meta-analysis. There was a small but statistically significant association between depression and lower subsequent attainment (pooled Fisher *z* = −0.19, 95% CI = −0.22 to −0.16, *I*^*2*^ = 62.9%). A total of 15 studies also reported an enduring effect after adjusting for various confounders. No statistically significant effect modifiers were identified. Social and school problems may mediate between depression and low attainment.

**Conclusion:**

Depression was associated with lower educational attainment, but further research is needed to establish mechanisms. Nonetheless, there is a clear need for mental health and educational support among children and adolescents with depression.

Globally, depression is a leading cause of illness and disability among adolescents.^1^ One study estimates that 3.2% of children and adolescents in the United States have depression, with prevalence increasing from 1.7% during middle-childhood (ages 6**−**11 years) to 6.1% during adolescence (ages 12**−**17 years), when key educational milestones often take place.^2^ Characterized by symptoms such as reduced energy, motivation, and concentration, depression in this age group could impair engagement, attendance, and performance at school.^3^, ^4^, ^5^

A recent meta-analysis conducted by Clayborne *et al.* investigated the association between adolescent depression and psychosocial outcomes during adulthood, including educational attainment.^6^ They concluded that adolescent depression was associated with reduced odds for completing high school and entering postgraduate education. However, these outcomes focused on emerging adulthood, and so were not able to capture the more immediate impact that depression is likely to have on attainment while children are still attending school. Indeed, an earlier systematic review by Riglin *et al.* found various internalizing disorders to negatively predict school grades.^7^ However, both reviews included studies that relied on self-reported attainment, which has demonstrated questionable validity; in particular, there is thought to be an association between depression symptoms and inaccuracy of self-reported grades.^8^, ^9^, ^10^, ^11^

In addition, both previous reviews had a broader focus on other exposure variables and outcome variables, such that there was limited scope to explore potential mechanisms for how depression during school might affect attainment. With multiple factors thought to be associated with mental health and educational outcomes,^12^ the pathway between depression and attainment potentially comprises a wide range of pupil-, parent-, teacher-, and school-level factors. It is therefore crucial that further review of this topic also seeks to synthesize evidence on effect modifiers and mediators to highlight potential targets for intervention.

To our knowledge, this is the first meta-analysis to focus exclusively on the longitudinal association between child and adolescent depression and subsequent educational attainment, as measured using school record data. The aim of this study was to provide a more robust estimate of this association, and to give a comprehensive overview of potential effect modifiers and mediators. This will guide future research and clinical interventions that aim to mitigate the long-term impact of mental health problems within educational settings.^13^ Based on previous studies, we hypothesized that depression would negatively predict subsequent educational attainment. As the first study to systematically review the pathway between these, we did not formulate any specific hypotheses regarding potential effect modifiers and mediators.

## Method

The protocol for this systematic review and meta-analysis can be viewed on PROSPERO at www.crd.york.ac.uk/PROSPERO/display_record.asp?ID=CRD42019123068, and has been published elsewhere.^14^ This review follows the Preferred Reporting Items for Systematic Reviews and Meta-Analyses (PRISMA) guidelines (see Table S1, available online).^15^

### Search Strategy and Inclusion Criteria

We included studies that (a) investigated and reported results on the relationship between depression diagnosis or depression symptoms (exposure variable, as measured using a standardized diagnostic measure or a named measurement instrument) and later educational attainment (outcome variable, as measured using academic or administrative records); (b) included participants all within the 4- to 18-year age range at exposure variable measurement (recruiting participants from a school year attended by children or adolescents within this age range was considered sufficient evidence of meeting this criterion); (c) made use of data from countries with compulsory education policies; (d) were longitudinal in design, with prospective data collection; (e) were original research published in a peer-reviewed journal; (f) were published in English; and (g) were available in full text.

We excluded studies that (a) only investigated internalizing symptoms more generally (for instance, encompassing symptoms of anxiety or stress) or other affective disorders (such as bipolar disorder); (b) recruited participants from postsecondary education settings; (c) only investigated school dropout, general intelligence, aptitude, or ability as the outcome; or (d) aimed to conduct or evaluate an intervention during the study period.

Because of the relationship under study, we were primarily interested in depression during the school years, so we selected the age range of 4 to 18 years to encompass the compulsory school age range in most countries. We also anticipated that studies would report a wide range of measures for educational attainment, and therefore did not impose any additional eligibility criteria relating to outcome measurement. The inclusion and exclusion criteria are discussed in further detail in the published protocol.^14^

Embase, PubMed, PsycINFO, the Education Resources Information Center, and the British Education Index were initially searched from inception up to November 13, 2018. The date range was unrestricted, but English language limits were applied because of a lack of resources for translation. Search terms were designed to capture studies that investigated the child and adolescent age group, educational attainment, and depression. Key-word searches were applied to titles and abstracts, and subject heading searches were tailored to each database’s thesaurus (see Supplement 1, available online). Backward and forward citation searching of included studies was conducted in Web of Science and Google Scholar, and reference lists of relevant reviews were also searched. Corresponding authors of included studies and experts in the field were contacted to identify additional citations.

Citations were managed and duplicates removed in EndNote, whereas screening and data extraction were tracked in Microsoft Excel. AW and HS independently screened titles, abstracts, and full texts obtained from electronic database searching, and a sample of those identified from citation searching following an initial screen by AW. Agreement was 90% following independent title and abstract screening, and 97% following independent full text screening. Disagreements were discussed and agreed upon between the 2 reviewers. The search was updated October 23, 2019; because of high agreement during the first screening, AW independently conducted the search update.

### Data Extraction and Analysis

Data were extracted from eligible studies using a structured coding form, into which AW and HS independently extracted country of study, participant age and gender, additional participant inclusion or exclusion criteria, exposure variable and outcome variable measurement, follow-up period, sample size, confounders investigated, effect modifiers and mediators investigated, and results used in meta-analysis. Authors were e-mailed for information that could not be found in the articles. Ethnicity was later extracted following feedback during peer review. Nationality, as distinct from ethnicity, was not extracted. AW and HS also independently conducted risk of bias assessment using an adapted version of the Newcastle−Ottawa Scale (NOS), which contains additional items to assess sample size and statistical tests, as has been used previously.^16^, ^17^, ^18^ Agreement was 80% following risk of bias assessment. AW independently conducted data extraction and risk of bias assessment resulting from the search update.

The effect sizes most frequently reported by included studies were correlation coefficients (bivariate associations between depression and attainment at a subsequent timepoint, having measured both on continuous scales). Therefore, correlation coefficients were pooled in random-effects meta-analysis, and results from multivariable analyses were summarized in a narrative synthesis. Correlation coefficients were transformed to the Fisher *z* and pooled in Stata version 15.0 using the metan command (see Supplement 2, available online).^19^, ^20^, ^21^, ^22^ Where studies reported multiple effect sizes that met the study eligibility criteria, the mean correlation coefficient and sample size were included in meta-analysis, as has been done previously.^23^ Heterogeneity was investigated using the Cochran *Q* and the *I*^*2*^ statistic,^24^ and publication bias was assessed with funnel plots and the Egger test.^25^

We additionally stratified the meta-analysis by studies that met at least half of the quality criteria specified on the NOS to assess risk of bias effects. Mean age at baseline (years) and follow-up period (months) were investigated as effect modifiers using random-effects meta-regression.^26^ If unavailable, mean age at baseline was estimated from the reported age range or school year. If 1 study reported multiple correlation coefficients relevant to different baseline ages, the coefficients for different ages were included separately in meta-regression. Where follow-up period was stated as a number of semesters, 1 semester was considered to equal 3 months. We conducted sensitivity analyses to investigate the various assumptions underpinning our meta-regressions, for instance, excluding studies in which the follow-up period was estimated from the number of semesters. Two other post hoc sensitivity analyses were conducted to investigate the effects of pooling different depression scales and studies conducted in different countries.

## Results

### Study Characteristics

**Figure 1:**
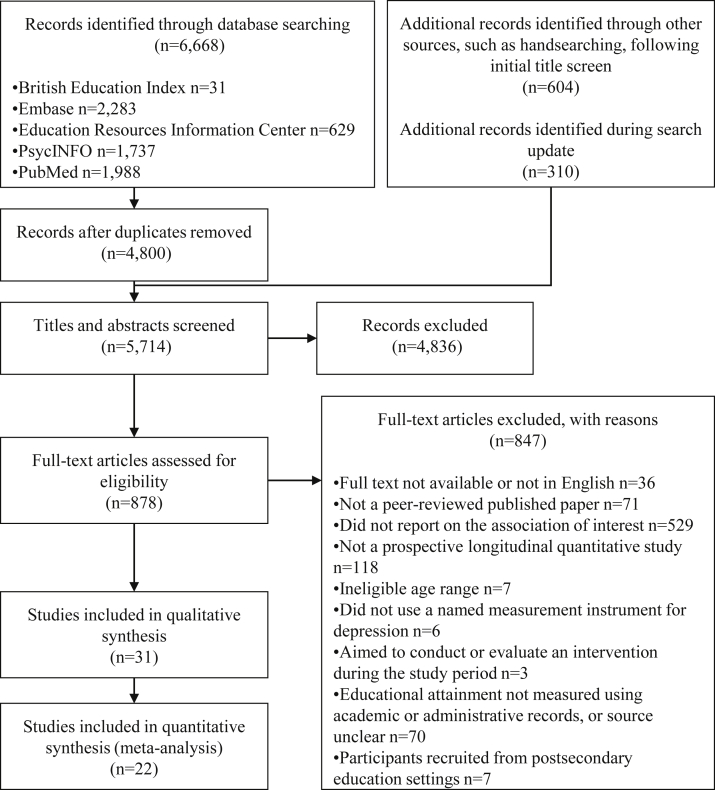
PRISMA Flow Diagram

In total, 5,714 studies were screened, of which 31 were eligible for inclusion (Figure 1, Table 1,^27^, ^28^, ^29^, ^30^, ^31^, ^32^, ^33^, ^34^, ^35^, ^36^, ^37^, ^38^, ^39^, ^40^, ^41^, ^42^, ^43^, ^44^, ^45^, ^46^, ^47^, ^48^, ^49^, ^50^, ^51^, ^52^, ^53^, ^54^, ^55^, ^56^, ^57^ Table S2, available online). Sample sizes ranged from n = 129 to n = 7,276.^47^^,^^54^ For those that reported age ranges, age at depression assessment ranged from 6 to 17 years.^35^^,^^37^^,^^45^^,^^54^ Gender was reasonably balanced, except in 1 study in which only 22% were male participants.^37^ All included studies sampled participants from schools, school districts, or the community. In accordance with the inclusion criteria, no associations were reported in which interventions were conducted during the study period; however, 2 samples had undergone interventions prior to the follow-up periods reported in this review.^36^^,^^41^ Almost all studies used scales and questionnaires to measure depression symptoms; only 1 study reported results from a diagnostic interview.^37^ Variants of the Children’s Depression Inventory (such as shortened or translated versions) were the most commonly used depression measurement instruments. Educational attainment was measured using grades and test scores across various subjects, and only 1 study investigated graduation from higher education as an outcome.^37^ The follow-up period ranged from less than one school term to 14 years.^31^^,^^37^

**Table 1:** Key Study Characteristics

First author, year, reference	Country	n	Ethnicity	Age and grade at depression measurement	Depression measurement	Attainment outcome(s)	Follow-up period	Total risk of bias score (maximum = 11)
Birchwood, 2012^27^	United Kingdom	324	Not reported	Mean = 15.55; range = 15−16; year 11	HADS (continuous)	GCSE points score; GSCE score weighted for number of GCSE entries (continuous)	1 school term	5
Chen, 2000^28^	China	431	Not reported	Mean = 11.58 (SD = 1.0); 6^th^ grade	CDI (continuous)	Chinese and mathematics scores (continuous)	2 y	8
Chen, 2013^29^	China	1155	Not reported	Mean = 9.33 (SD = 0.67); 3^rd^ grade	CDI (continuous)	Chinese, mathematics, and English scores (continuous)	1 y	7
Chen, 2019^30^	China	1430	Not reported	Mean = 15.43; 7^th^ and 10^th^ grade	CDI (continuous)	Chinese, mathematics, and English scores (continuous)	1 y	9
Da Fonseca, 2009^31^	France	353	Not reported	Mean = 13.25 (SD = 4.3); range = 11−16	CDI (continuous)	Mathematics grade (continuous)	<1 school term	4
Davies, 2018^32^	United Kingdom	4398	Not reported	Range = 12	SMFQ (continuous and binary)	Achieving 5 or more A∗−C grades at GCSE (binary); achieving 3 or more A∗−C grades at A level (binary)	4 and 6 y	7
Denault, 2009^33^	Canada	362	Majority White; 3% Black; 1% Asian; 3% Latino; 3% Arabic	Mean = 12.38 (SD = 0.42); 7^th^−9^th^ grade	CDI (continuous)	Mathematics and French scores (continuous)	1−3 y	7
Fu, 2018^34^	China	336	Not reported	Mean = 14.08 (SD = 0.58); 7^th^−8^th^ grade	CDI (continuous)	Chinese, mathematics and English scores (continuous)	1 y	7
Hood, 2017^35^	Australia	244	‘Ethnically diverse school’	Mean = 13.6 (SD = 1.24); range = 11−17; years 7−10	DASS (continuous)	Average grade in “all subjects” (continuous)	1 semester	5
Ialongo, 2001^36^	United States of America	625	66% African American; 32% European American; 2% Native American, Asian American, and Hispanic American	Range = 9−10^a^; 4^th^ grade	MFQ-PSF (binary)	Overall GPA of C or worse (binary)	1−2 y	9
Jonsson, 2010^37^	Sweden	588	Not reported	Mean = 16.44 (SD = 0.63)^a^; range = 16-17; first year of upper secondary school	BDI, CES-D for children, *DICA-R-A* (binary)	Final GPA in upper secondary school (continuous); attained an upper secondary school diploma by age 20 y (binary); graduated from higher education by age 30 y (binary); graduated given that they entered higher education (binary)	2−14 y	10
Kim, 2013^38^	United States of America	379	Chinese American (mostly from Hong Kong or southern provinces of China)	Mean = 13.04 (SD = 0.73); range = 12−15; 7^th^−8^th^ grade	CES-D (continuous)	GPA (excluding physical education courses) and California Standards Test scores in mathematics and English (continuous)	4 y	6
Kim, 2015^39^	United States of America	350	Chinese American (mostly from Hong Kong or southern provinces of China; <10 families from Taiwan)	Mean = 13.03 (SD = 0.73); range = 12−15; 7^th^−8^th^ grade	CES-D (continuous)	GPA (excluding physical education courses) (continuous)	4 y	6
Kingery, 2011^40^	United States of America	365	99% Caucasian	Mean = 11.17; 5^th^ grade	CDI (continuous)	English, science, social studies, and mathematics grades (continuous)	6 mo	5
Lepore, 2013^41^	United States of America	498	43% White/Caucasian; 24% Latino/Latina; 24% Black/African American; 9% other	Mean = 12.8 (SD = 0.44); 7^th^ grade	CDI (continuous)	Cumulative GPA across English, mathematics, social studies, and science (continuous)	6 mo	6
Liu, 2018^42^	China	945	Not reported	Mean = 10.16 (SD = 0.17); 4^th^−5^th^ grade	CDI (continuous)	Chinese, mathematics and English grades (continuous)	1 and 2 y	8
Luthar, 1995^43^	United States of America	138	40% African American; 27% Hispanic; 9% Asian; 8% Mixed heritage	Mean = 15.2 (SD = 1.0); 9^th^ grade	CDI (continuous)	Mean grade across 4 academic courses (continuous)	6 mo	7
McLeod, 2012^44^	United States of America	4701	54% White; 19% African American; 17% Latino/Latina; 7% Asian; 1% Native American; 1% other	Mean = 16.07 (SD = 1.13); 9^th^−12^th^ grade	CES-D (binary)	Post−Wave I high school GPA (continuous)	1−3 y^a^	8
Morales, 2006^45^	United States of America	2745	42% African American; 45% Latino; 13% non-Hispanic White	Range = 6−11; 1^st^−4^th^ grade	CBCL-TRF (continuous)	Reading and mathematics scores from Iowa Test of Basic Skills (continuous)	2 y	5
Nishina, 2005^46^	United States of America	1526	45% Latino (primarily Mexican and Central American); 26% African American; 11% Asian; 9% European American; 8% mixed	6^th^ grade	CDI (continuous)	GPA across all classes (continuous)	1 semester	5
Pate, 2017^47^	United States of America	7276	60% White; 20% Black; 6% Asian/Pacific Islander; 1% Native American; 12% other/multiracial	Mean = 14.71 (SD = 1.09); range = 13−16; 7^th^−12^th^ grade	CES-D (continuous)	Cumulative GPA across all waves (continuous)	7 y	9
Riglin, 2013^48^	United Kingdom	202	Not reported	Mean = 11.25 (SD = 0.44); year 7	SMFQ (continuous)	English, mathematics, and science scores (continuous)	2 school terms	7
Rockhill, 2009^49^	United States of America	521	44% Caucasian/White; 26% African; 26% Asian; 4% Native American; 10% Hispanic	Mean = 12.0 (SD = 0.41)^b^; 6^th^ grade	MFQ (categorical)	Cumulative GPA (continuous)	6 mo	6
Rothon, 2009^50^	United Kingdom	1636	26% Bangladeshi; 18% White UK; 9% Asian Indian; 7% Pakistani; 6% Black Caribbean; 11% Black African; 4% Black British; 18% other	Range = 13−14; year 9	SMFQ (continuous)	Achieving 5 or more A∗−C grades at GCSE (binary)	1−3 y	9
Rothon, 2011^51^	United Kingdom	2499	26% Bangladeshi; 20% White UK; 9% Asian Indian; 7% Pakistani; 6% Black Caribbean; 10% Black African; 22% other	Range = 13−14; year 9	SMFQ (binary)	Achieving 5 or more A∗−C grades at GCSE (binary)	2 y	7
Schwartz, 2005^52^	United States of America	199	36% Hispanic; 26% European American; 7% Asian American; 2% African American; 23% other (such as mixed); 6% unclassified	Mean = 9.02 (SD = 0.57); range = 8.01−10.57; 3^rd^−4^th^ grade	CDI (continuous)	GPA and scores on mathematics and reading subscales of the Stanford Achievement Test−Ninth Edition (continuous)	1 y	6
Shahar, 2006^53^	United States of America	460	49% Non-Hispanic White; 26% Hispanic; 22% African American; 3% other	Range = 11−14^b^; 6^th^−7^th^ grade	BDI (continuous)	GPA in English/reading, mathematics, social studies, and science (continuous)	1 y	7
Steele, 2000^54^	United States of America	129	African American	Mean = 8.78 (SD = 1.69); Range = 6-11	CDI (continuous)	GPA across each academic subject (continuous)	12−14 mo	8
Wang, 2014^55^	United States of America	935	53% European American; 40% African American; 7% biracial/other	10^th^ grade	CDI (continuous)	English, mathematics, science, and social sciences grades (continuous)	1 y minimum	9
Weidman, 2015^56^	United States of America	130	92% White; 4% Black; 2% Hispanic; 2% Asian	Mean = 11.17 (SD = 0.38); 6^th^−9^th^ grade	CDI (continuous)	GPA in English, mathematics, history/social studies, and science (continuous)	Up to 4 y	8
Zhang, 2019^57^	China	648	Nearly all Chinese Han	Mean = 11.18 (SD = 0.35); range = 95% of children aged 11.15−11.21^a^; 5^th^−8^th^ grade	CDI (continuous)	Chinese, mathematics, and English scores (continuous)	1 y	9

Note: BDI = Beck Depression Inventory; CBCL-TRF = Child Behavior Checklist−Teacher Report Form; CDI = Children's Depression Inventory; CES-D = Center for Epidemiologic Studies of Depression Scale; DASS = Depression Anxiety Stress Scale; *DICA-R-A* = Diagnostic Interview for Children and Adolescents in the revised form according to DSM-III-R for adolescents; GCSE = General Certificate of Secondary Education; GPA = Grade Point Average; HADS = Hospital Anxiety and Depression Scale; MFQ = Mood and Feelings Questionnaire; MFQ-PSF = Mood and Feelings Questionnaire−Parent Short Form; SMFQ = Short Mood and Feelings Questionnaire.

a Information provided or confirmed by the author.

b Estimated from another article that made use of the same or similar data, as recommended by the author.

### Risk of Bias

The overall quality of included studies was good; the mean NOS score was 7/11, and only 6 studies met less than half of the assessed quality criteria (Table 1; Table S3, available online). However, the representativeness of samples was mixed, and none of the included studies reported power analyses or otherwise justified their sample sizes, such that some of the smaller studies may have been underpowered. Most of the studies controlled for confounders in their design or analysis, and several also adjusted for educational attainment at baseline. Many studies did not fully report results from statistical tests, for instance, omitting named effect estimates, *p* values, or measures of precision if appropriate (such as standard errors or confidence intervals).

### Meta-analyses

A total of 22 studies were included in meta-analysis (see Tables S4 and S5, available online). All of the studies combined in meta-analysis reported correlation coefficients between depression symptoms and attainment, as measured using continuous scales, and none of these studies reported using overlapping samples. The remaining 9 studies did not report bivariate associations between continuous measures of depression and attainment at a subsequent timepoint, and so could not be combined in this meta-analysis. Regrettably, they also did not report analyses that were sufficiently comparable to each other to combine into a separate meta-analysis.

**Figure 2:**
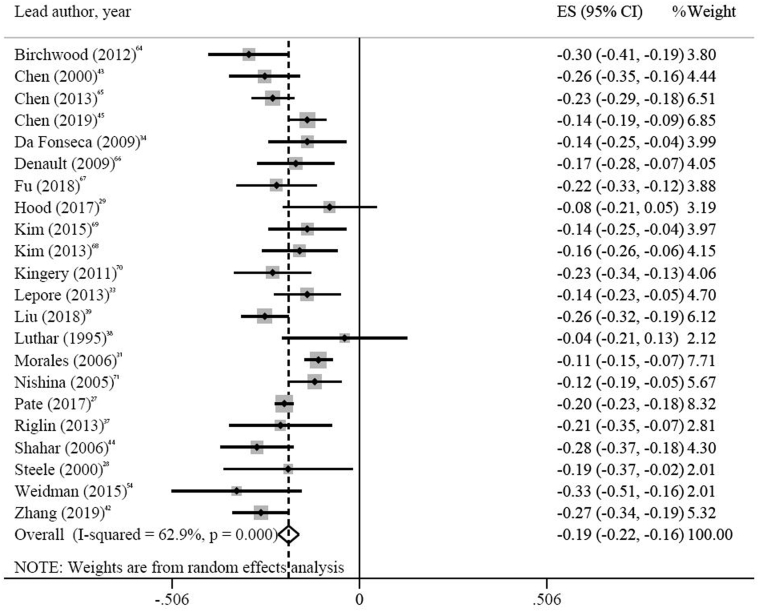
Meta-analysis of Correlation Coefficients Between Depression and Educational Attainment

We found a statistically significant negative association between depression and attainment (pooled Fisher *z* = −0.19, 95% CI = −0.22 to −0.16, *z* = 13.32, *p* < .001) (Figure 2). This is equivalent to a pooled correlation coefficient of *r* = −0.19 (see Supplement 3, available online), a small effect indicating that depression symptoms accounted for 3.6% of the variance in attainment.^20^^,^^58^ There was moderate and statistically significant heterogeneity between the studies [*I*^*2*^ = 62.9%; *Q*(21) = 56.66, *p* <.001], and no evidence of publication bias (see Figure S1, available online; Egger test *p* = .630). Meta-regression did not show any statistically significant effects of follow-up period (*p* = .934) or age at baseline (*p* = .989), and this remained the case following sensitivity analyses for the various assumptions of meta-regression (results available from authors upon request). Stratification by study quality (scores above and below 50% on the risk of bias assessment) resulted in similar pooled effect sizes (see Table S6, available online). Other than changes in heterogeneity as might be expected, post hoc sensitivity analyses also yielded similar small but significant pooled effect sizes.

### Narrative Synthesis of Potential Effect Modifiers, Mediators, and Confounders

In all, 22 of the included studies investigated confounding, effect modification, or mediation (see Table S7, available online). A wide range of confounders were considered, such as sociodemographics, other mental health and school problems, and parent and family characteristics. A total of 15 studies still found a statistically significant, negative association between depression and attainment after adjusting for all confounders. Moreover, although our risk of bias analysis showed that only a minority of studies adjusted for educational attainment at baseline, thus limiting scope for inferring the relationship’s direction, 8 of the 12 included studies that did adjust for attainment at an earlier timepoint found a statistically significant association. One study found a weak but significant positive association between depression at age 12 years and performance on Advanced Level examinations (typically obtained at age 18 years in the United Kingdom), even after adjusting for various confounders. The authors attribute this to the Short Mood and Feelings Questionnaire detection of traits relating to neuroticism.^32^ This finding was not replicated for performance on General Certificate of Secondary Education examinations (typically obtained at age 16 years), which may also indicate differential impacts by age or school stage.

The most commonly investigated effect modifier was gender, although findings were inconclusive, with some studies finding a significant association only in male participants,^36^^,^^48^ others only in female participants,^43^ and still more finding no effect of gender.^28^^,^^41^^,^^42^^,^^44^^,^^50^^,^^53^^,^^54^^,^^57^ Some found effects of gender that were dependent on the level of other variables such as baseline attainment,^28^ how attainment was measured,^37^ and level of self-criticism.^53^ There was also some evidence that school connectedness and ethnicity moderated the association between depression and attainment; but, as with gender, the modifying effect of ethnic variation was inconsistent.^47^^,^^41^^,^^50^ One study also investigated school year as an effect modifier, but did not find a significant effect.^30^

Finally, 3 of the included studies investigated possible mediators on the pathway between depression and attainment. These provided some evidence that the association may be partly mediated by peer victimization,^42^ school connectedness,^47^ social competence, and social support.^49^ Taken together, these findings are suggestive that higher levels of depression symptoms may be associated with lower levels of social support (as measured in a variety of ways), which in turn may lead to lower attainment. Social support may therefore be a mechanism underlying the negative association between depression and attainment observed in our meta-analysis.

## Discussion

This systematic review and meta-analysis provides comprehensive evidence for a small but significant longitudinal association between child and adolescent depression symptoms and lower educational attainment, which persisted despite adjustment for a number of potential confounders. The findings also demonstrate that peer victimisation, school connectedness and social support factors may mediate between depression and low attainment. We were unable to conclude whether gender and ethnicity were modifiers on the depression-attainment pathway. Finally, we found sample age and duration of the longitudinal follow-up were not significant effect modifiers.

The recent review by Clayborne *et al.*^6^ investigated the association between adolescent depression and educational attainment at emerging adulthood. By extending our review to include studies that measured educational attainment *during* the school years, we identified 29 studies not eligible for inclusion by Clayborne *et al.* Most of these measured academic performance while participants were still of school age, thereby emphasizing that the negative association between depression and attainment is not merely a problem that presents itself by the completion of secondary education and engagement in higher education. Timely detection and intervention during school is therefore critical. Our findings also update an earlier review by Riglin *et al.*, again identifying 28 studies on the association that they did not capture.^7^ Moreover, we limited our review to studies that measured performance from school records, thereby overcoming known issues with the validity of self-reported attainment, and providing a more robust measure of the association than the previous reviews.^8^^,^^9^

Unlike these previous reviews, we conducted a narrative synthesis of various effect modifiers and mediators, and demonstrated that there remains a significant gap in available evidence to build a robust pathway model between depression and attainment. In particular, findings relating to gender and ethnicity were varied and inconclusive, and although social support emerged as a potential mediator, it has been inconsistently examined. Given that our results suggest that child- and adolescent-onset depression is a weak but highly modifiable risk factor for low attainment, a fuller understanding of the relationship between depression and attainment is critical. Fuller analyses of the causal pathway should combine the range of potential modifiers and mediators, such as key predictors of attainment and other school factors associated with depression. For instance, a range of other sociodemographic characteristics have been associated with attainment and mental health, such as socioeconomic status and relative age in the school year.^59^^,^^60^ Other candidate variables in the pathway might include parent and teacher support,^61^^,^^62^ school absence,^4^ pupil engagement,^63^ school involvement,^3^ and academic self-efficacy.^64^ A better understanding of how certain aspects of depression may affect attainment would also be beneficial: for example, whether specific symptoms or the duration of experiencing symptoms has a particularly negative impact on attainment.

The role of externalizing symptoms in the association is also unclear. A previous narrative review suggested that there was no direct association between internalizing problems (ie, depression and anxiety) and academic outcomes after adjusting for baseline attainment, and that externalizing problems play a more critical role.^65^ To the contrary, we identified multiple studies in which the association between depression and attainment was robust to baseline adjustments, but the role of externalizing problems does remain uncertain. McLeod *et al.* concluded that behavior problems were a stronger predictor of attainment than depression, but acknowledged that some externalizing problems such as substance use might be coping responses for children with depression, and may thus be a mediating factor.^44^ However, Rockhill *et al.* concluded that depression that is comorbid with conduct problems is a stronger predictor of attainment than either problem in isolation, such that the presence of externalizing comorbidities might be considered an effect modifier in the association between depression and attainment.^49^ This area would benefit from further research.

This review has a number of strengths. We conducted a thorough search of both health and education databases, and included studies that used named and established depression measures and administrative attainment data to produce as robust an estimate of the association as possible. In part due to our robust inclusion criteria, the risk of bias of included studies was generally low. We also focused on depression symptoms to the exclusion of broader constructs such as “internalizing symptoms” or “psychological distress.” These measures often include symptoms of anxiety, which Weidman *et al.* argue is motivationally and cognitively distinct from depression, such that its inclusion might obscure the effects of depression symptoms.^56^

This review also has some limitations. Our focus on longitudinal studies with prospective data collection aids inference of a causal direction, but scope for detecting an independent association was limited by our inability to pool effect sizes that adjusted for covariates including prior attainment in meta-analysis. Many included studies did not conduct comparable multivariable analyses, adjusting for a wide range of covariates and variable reporting of results, or did not report their results fully enough to incorporate them into the meta-analysis. Bivariate associations were therefore pooled to maximize the number of studies captured in the meta-analysis.

Our age range for depression measurement (4−18 years) was designed to encompass the compulsory school age range in most countries, but therefore captures a wide age range and a number of heterogenous developmental periods. We also did not limit inclusion of studies based on length of follow-up in order to investigate its potential effects through meta-regression. Therefore, some of the included studies have a very short follow-up period between depression assessment and outcome ascertainment, further limiting causal inference. However, the majority of included studies included a follow-up period of at least 1 year. In addition, the meta-regression suggested that the strength of the association between depression and attainment was robust to follow-up duration.

There was no evidence of publication bias, but we were able to include only studies published in the English language. Research in non−English-language journals or regional databases, and particularly research conducted in low- and middle-income countries, might therefore be missed, limiting generalizability to countries not captured by this study.^66^ We also pooled estimates from a wide variety of countries and cultures, despite substantial variation in educational systems. However, we note that our stratified meta-analysis found similar effects after pooling results within the United Kingdom, United States, and China only.

The included studies were significantly heterogenous, suggesting a role for unexplored effect modifiers. For instance, we were unable to meta-analyze modifying effects of gender because of the limited reporting of correlation coefficients stratified by gender. Finally, the majority of included studies investigated depression symptoms as the exposure variable, rather than depression diagnosis, such that the clinical significance of the reported associations remains unclear.

Lower school performance can have a lasting impact throughout the life course, predicting unemployment, homelessness, poor health, and suicide attempts.^67^, ^68^, ^69^, ^70^ Indeed, our finding that depression can have an impact on grades during the school career appear to extend into educational outcomes in adulthood, as demonstrated by Clayborne *et al.*^6^ In turn, education is associated with long-term economic growth, and therefore has implications at the societal level.^71^ Our findings should therefore serve to motivate the development of evidence-based interventions for children and adolescents with depression, and future work should investigate whether detection and treatment of depression symptoms in pupil populations leads to improved educational outcomes.^72^^,^^73^ Observational or trial-based research that develops a pathway model between depression and attainment could inform these efforts and help to reduce the attainment gap relating to child and adolescent depression. It should also be noted that we have focused on the pathway from depression to attainment, and comparatively few of our included studies measured depression and attainment at multiple timepoints, which would be an important inclusion in future studies on this topic. Indeed, some evidence suggests that the relationship may be bi-directional.^56^ A recent genome-wide association meta-analysis found a possible relationship between genetic variation in educational attainment and major depressive disorder.^74^ As a result, children and adolescents might enter a vicious cycle of poor mental health and school performance, such that interventions targeting depression and learning behaviors may both be beneficial. With this in mind, families, clinicians, and educators should engage in comprehensive approaches that can address modifiable, shared precursors for both depression and poor attainment (such as school absence),^4^ rather than targeting single risk factors in isolation.

In conclusion, we conducted a comprehensive systematic review and meta-analysis of the association between depression and subsequent educational attainment in children and adolescents, and found evidence for a small but significant negative association. Going forward, the research landscape would benefit from further research on the pathway between depression and educational attainment. Nonetheless, the need for mental health and educational support among children and adolescents who struggle with depression is clear.
