# Supplementary material for: Systematic Review and Meta-analysis: The Association Between Child and Adolescent Depression and Later Educational Attainment
Source: J Am Acad Child Adolesc Psychiatry. 2021 Jan;60(1):105–18. doi: 10.1016/j.jaac.2020.10.008 (PMC7779367; doi:10.1016/j.jaac.2020.10.008)
Supplement: Supplemental Material [file mmc1.docx]

| **Section/topic** | **#** | **Checklist item** | **Reported on page #** |
| --- | --- | --- | --- |
| **TITLE** | | |  |
| Title | 1 | Identify the report as a systematic review, meta-analysis, or both. | Title page |
| **ABSTRACT** | | |  |
| Structured summary | 2 | Provide a structured summary including, as applicable: background; objectives; data sources; study eligibility criteria, participants, and interventions; study appraisal and synthesis methods; results; limitations; conclusions and implications of key findings; systematic review registration number. | 1-2 |
| **INTRODUCTION** | | |  |
| Rationale | 3 | Describe the rationale for the review in the context of what is already known. | 3-4 |
| Objectives | 4 | Provide an explicit statement of questions being addressed with reference to participants, interventions, comparisons, outcomes, and study design (PICOS). | 3-4 |
| **METHODS** | | |  |
| Protocol and registration | 5 | Indicate if a review protocol exists, if and where it can be accessed (e.g., Web address), and, if available, provide registration information including registration number. | 4 |
| Eligibility criteria | 6 | Specify study characteristics (e.g., PICOS, length of follow-up) and report characteristics (e.g., years considered, language, publication status) used as criteria for eligibility, giving rationale. | 5 |
| Information sources | 7 | Describe all information sources (e.g., databases with dates of coverage, contact with study authors to identify additional studies) in the search and date last searched. | 6 |
| Search | 8 | Present full electronic search strategy for at least one database, including any limits used, such that it could be repeated. | Supplement |
| Study selection | 9 | State the process for selecting studies (i.e., screening, eligibility, included in systematic review, and, if applicable, included in the meta-analysis). | 6 |
| Data collection process | 10 | Describe method of data extraction from reports (e.g., piloted forms, independently, in duplicate) and any processes for obtaining and confirming data from investigators. | 6-7 |
| Data items | 11 | List and define all variables for which data were sought (e.g., PICOS, funding sources) and any assumptions and simplifications made. | 6-7 |
| Risk of bias in individual studies | 12 | Describe methods used for assessing risk of bias of individual studies (including specification of whether this was done at the study or outcome level), and how this information is to be used in any data synthesis. | 7 |
| Summary measures | 13 | State the principal summary measures (e.g., risk ratio, difference in means). | 7 |
| Synthesis of results | 14 | Describe the methods of handling data and combining results of studies, if done, including measures of consistency (e.g., I^2^) for each meta-analysis. | 7 |

| **Section/topic** | **#** | **Checklist item** | **Reported on page #** |
| --- | --- | --- | --- |
| Risk of bias across studies | 15 | Specify any assessment of risk of bias that may affect the cumulative evidence (e.g., publication bias, selective reporting within studies). | 7 |
| Additional analyses | 16 | Describe methods of additional analyses (e.g., sensitivity or subgroup analyses, meta-regression), if done, indicating which were pre-specified. | 7-8 |
| **RESULTS** | | |  |
| Study selection | 17 | Give numbers of studies screened, assessed for eligibility, and included in the review, with reasons for exclusions at each stage, ideally with a flow diagram. | 9 |
| Study characteristics | 18 | For each study, present characteristics for which data were extracted (e.g., study size, PICOS, follow-up period) and provide the citations. | 10 |
| Risk of bias within studies | 19 | Present data on risk of bias of each study and, if available, any outcome level assessment (see item 12). | 10-11 |
| Results of individual studies | 20 | For all outcomes considered (benefits or harms), present, for each study: (a) simple summary data for each intervention group (b) effect estimates and confidence intervals, ideally with a forest plot. | 12-13 |
| Synthesis of results | 21 | Present results of each meta-analysis done, including confidence intervals and measures of consistency. | 12-13 |
| Risk of bias across studies | 22 | Present results of any assessment of risk of bias across studies (see Item 15). | 13 |
| Additional analysis | 23 | Give results of additional analyses, if done (e.g., sensitivity or subgroup analyses, meta-regression [see Item 16]). | 13 |
| **DISCUSSION** | | |  |
| Summary of evidence | 24 | Summarize the main findings including the strength of evidence for each main outcome; consider their relevance to key groups (e.g., healthcare providers, users, and policy makers). | 15-20 |
| Limitations | 25 | Discuss limitations at study and outcome level (e.g., risk of bias), and at review-level (e.g., incomplete retrieval of identified research, reporting bias). | 18-19 |
| Conclusions | 26 | Provide a general interpretation of the results in the context of other evidence, and implications for future research. | 15-20 |
| **FUNDING** | | |  |
| Funding | 27 | Describe sources of funding for the systematic review and other support (e.g., supply of data); role of funders for the systematic review. | Title page |

*Note:*  From Moher D, Liberati A, Tetzlaff J, Altman DG, The PRISMA Group (2009). Preferred Reporting Items for Systematic Reviews and Meta-Analyses: The PRISMA Statement. PLoS Med 6(7): e1000097. doi:10.1371/journal.pmed1000097

For more information, visit: **www.prisma-statement.org**.

**Supplement 1: Database search terms**

**Embase**

| **#** | **Search terms** |
| --- | --- |
| 1 | exp adolescent/ OR exp adolescence/ OR exp child/ OR exp childhood/ OR child*.tw OR adolescen*.tw OR teenag*.tw OR youth*.tw OR (young adj (people or person)).tw |
| 2 | Limit 1 to english language |
| 3 | exp academic achievement/ OR exp outcome of education/ OR ((academic or educational or school or classroom) adj (achievement or performance or attainment or success or failure)).tw |
| 4 | Limit 3 to english language |
| 5 | exp depression/ OR depressi*.tw |
| 6 | Limit 5 to english language |
| 7 | #2 AND #4 AND #6 |

Note: exp [search term]/ denotes exploding a subject heading; .tw denotes searching for a key word in the title, abstract and drug trade name; * denotes truncation

**PsycINFO**

| **#** | **Search terms** |
| --- | --- |
| 1 | child*.tw OR adolescen*.tw OR teenag*.tw OR youth*.tw OR (young adj (people or person)).tw |
| 2 | Limit 1 to english language |
| 3 | exp academic achievement/ OR exp academic failure/ OR exp educational attainment level/ OR exp grade level/ OR ((academic or educational or school or classroom) adj (achievement or performance or attainment or success or failure)).tw |
| 4 | Limit 3 to english language |
| 5 | exp affective disorders/ OR depressi*.tw |
| 6 | Limit 5 to english language |
| 7 | #2 AND #4 AND #6 |

Note: exp [search term]/ denotes exploding a subject heading; .tw denotes searching for a key word in the table of contents, title, abstract, and key concepts; * denotes truncation

**PubMed**

|  | **Search terms** |
| --- | --- |
|  | adolescent[MESH] OR child[MESH] OR child*[Title/Abstract] OR adolescen*[Title/Abstract] OR teenag*[Title/Abstract] OR youth*[Title/Abstract] OR “young people”[Title/Abstract] OR “young person”[Title/Abstract] |
| AND | academic performance[MESH] OR educational status[MESH] OR “academic achievement”[Title/Abstract] OR “academic performance”[Title/Abstract] OR “academic attainment”[Title/Abstract] OR “academic success”[Title/Abstract] OR “academic failure”[Title/Abstract] OR “educational achievement”[Title/Abstract] OR “educational performance”[Title/Abstract] OR “educational attainment”[Title/Abstract] OR “educational success”[Title/Abstract] OR “educational failure”[Title/Abstract] OR “school achievement”[Title/Abstract] OR “school performance”[Title/Abstract] OR “school attainment”[Title/Abstract] OR “school success”[Title/Abstract] OR “school failure”[Title/Abstract] OR “classroom achievement”[Title/Abstract] OR “classroom performance”[Title/Abstract] OR “classroom success”[Title/Abstract] |
| AND | depression[MESH] OR depressive disorder[MESH] OR depressi*[Title/Abstract] |

Note: [MESH] denotes a subject heading search; [Title/Abstract] denotes searching for a key word in the title or abstract; * denotes truncation. An English language filter was applied to this search. Key words “classroom attainment”[Title/Abstract] and “classroom failure”[Title/Abstract] were piloted, but removed from the final search because they produced no results.

**British Education Index**

|  | **Search terms** |
| --- | --- |
|  | ((DE "CHILDREN" OR DE "ABUSED children" OR DE "ADOPTED children" OR DE "BIRTH order" OR DE "BLACK children" OR DE "BOYS" OR DE "CHILD development" OR DE "CHILDREN as teachers" OR DE "CHILDREN of attention-deficit-disordered parents" OR DE "CHILDREN of divorced parents" OR DE "CHILDREN of foreign workers" OR DE "CHILDREN of immigrants" OR DE "CHILDREN of migrant laborers" OR DE "CHILDREN of minorities" OR DE "CHILDREN of older parents" OR DE "CHILDREN of parents with disabilities" OR DE "CHILDREN of school principals" OR DE "CHILDREN of single parents" OR DE "CHILDREN of teenage mothers" OR DE "CHILDREN of the rich" OR DE "CHILDREN of unmarried parents" OR DE "CHILDREN of working parents" OR DE "CHILDREN'S television programs" OR DE "CITY children" OR DE "EXCEPTIONAL children" OR DE "FOSTER children" OR DE "GIRLS" OR DE "GRANDCHILDREN" OR DE "HANDICRAFT for children" OR DE "HOMELESS children" OR DE "MENTALLY ill children" OR DE "PLAYMATES" OR DE "POOR children" OR DE "PRESCHOOL children" OR DE "PROBLEM children" OR DE "REFUGEE children" OR DE "RELIGIOUS education of children" OR DE "SCHOOL children") OR (DE "ADOLESCENCE")) OR (DE "YOUTH" OR DE "AT-risk youth" OR DE "BISEXUAL youth" OR DE "BLACK youth" OR DE "JUVENILE delinquents" OR DE "LGBT youth" OR DE "MENTALLY ill youth" OR DE "MINORITY youth" OR DE "PROBLEM youth" OR DE "RELIGIOUS education of young people" OR DE "SCHOOL dropouts" OR DE "TEENAGERS" OR DE "URBAN youth" OR DE "YOUNG adults" OR DE "YOUTH with disabilities") OR TI (“child*” OR “adolescen*” OR “teenag*” OR “youth*” OR “young people” OR “young person”) OR AB (“child*” OR “adolescen*” OR “teenag*” OR “youth*” OR “young people” OR “young person”) |
| AND | ((DE "ACADEMIC achievement" OR DE "ACADEMIC overachievement" OR DE "ACADEMIC underachievement" OR DE "ACHIEVEMENT gap" OR DE "ACHIEVEMENT in science" OR DE "ACHIEVEMENT tests" OR DE "BIRTH date effect (Academic achievement)" OR DE "COMPOSITION (Language arts) achievement" OR DE "COMPREHENSIVE examinations" OR DE "EDUCATIONAL attainment" OR DE "GRADUATION (Education)" OR DE "MOTIVATION in education" OR DE "PERSONALITY &amp; academic achievement" OR DE "PREDICTION of scholastic success") OR (DE "SCHOOL failure")) OR (DE "GRADING & marking (Students)" OR DE "ABILITY grouping (Education)" OR DE "ACADEMIC dissertations -- Evaluation" OR DE "COLLEGE credits -- Outside work" OR DE "EDUCATIONAL tests &amp; measurements -- Scaling" OR DE "ESTIMATED true score" OR DE "GRADE advancement" OR DE "GRADE inflation" OR DE "GRADE point average" OR DE "OUT-of-level testing" OR DE "PASS-fail grading system" OR DE "PROMOTION (School)" OR DE "SCHOOL credits" OR DE "SCHOOL credits -- Outside work" OR DE "SCHOOL grade placement") OR TI (“academic achievement” OR “academic performance” OR “academic attainment” OR “academic success” OR “academic failure” OR “educational achievement” OR “educational performance” OR “educational attainment” OR “educational success” OR “educational failure” OR “school achievement” OR “school performance” OR “school attainment” OR “school success” OR “school failure” OR “classroom achievement” OR “classroom performance” OR “classroom success” OR “classroom failure”) OR AB (“academic achievement” OR “academic performance” OR “academic attainment” OR “academic success” OR “academic failure” OR “educational achievement” OR “educational performance” OR “educational attainment” OR “educational success” OR “educational failure” OR “school achievement” OR “school performance” OR “school attainment” OR “school success” OR “school failure” OR “classroom achievement” OR “classroom performance” OR “classroom attainment” OR “classroom success” OR “classroom failure”) |
| AND | DE "MENTAL depression" OR DE "DEPRESSION in college students" OR TI “depressi*” OR AB “depressi*” |

Note: DE denotes a subject heading search; TI denotes searching for a key word in the title; AB denotes searching for a key word in the abstract; * denotes truncation. The key word search TI “classroom attainment” was piloted, but removed from the final search because it produced no results.

**Education Resources Information Center**

|  | **Search terms** |
| --- | --- |
|  | ((((DE "Children" OR DE "African American Children" OR DE "Grandchildren" OR DE "Hospitalized Children" OR DE "Latchkey Children" OR DE "Migrant Children" OR DE "Minority Group Children" OR DE "Preadolescents" OR DE "Young Children") OR (DE "Adolescents")) OR (DE "Early Adolescents")) OR (DE "Late Adolescents")) OR (DE "Youth" OR DE "Disadvantaged Youth" OR DE "Out of School Youth" OR DE "Rural Youth" OR DE "Urban Youth") OR TI (“child*” OR “adolescen*” OR “teenag*” OR “youth*” OR “young people” OR “young person”) OR AB (“child*” OR “adolescen*” OR “teenag*” OR “youth*” OR “young people” OR “young person”) |
| AND | (((DE "Achievement" OR DE "Academic Achievement" OR DE "African American Achievement" OR DE "Graduation" OR DE "High Achievement" OR DE "Knowledge Level" OR DE "Low Achievement" OR DE "Mathematics Achievement" OR DE "Overachievement" OR DE "Reading Achievement" OR DE "Scholarship" OR DE "Science Achievement" OR DE "Underachievement" OR DE "Writing Achievement") OR (DE "Failure" OR DE "Academic Failure")) OR (DE "Grades (Scholastic)" OR DE "Grade Inflation" OR DE "Grade Point Average")) OR (DE "Student Evaluation" OR DE "Curriculum Based Assessment" OR DE "Nongraded Student Evaluation" OR DE "Progress Monitoring") OR TI (“academic achievement” OR “academic performance” OR “academic attainment” OR “academic success” OR “academic failure” OR “educational achievement” OR “educational performance” OR “educational attainment” OR “educational success” OR “educational failure” OR “school achievement” OR “school performance” OR “school attainment” OR “school success” OR “school failure” OR “classroom achievement” OR “classroom performance” OR “classroom success” OR “classroom failure”) OR AB (“academic achievement” OR “academic performance” OR “academic attainment” OR “academic success” OR “academic failure” OR “educational achievement” OR “educational performance” OR “educational attainment” OR “educational success” OR “educational failure” OR “school achievement” OR “school performance” OR “school attainment” OR “school success” OR “school failure” OR “classroom achievement” OR “classroom performance” OR “classroom attainment” OR “classroom success” OR “classroom failure”) |
| AND | DE "Depression (Psychology)" OR TI “depressi*” OR AB “depressi*” |

Note: DE denotes a subject heading search; TI denotes searching for a key word in the title; AB denotes searching for a key word in the abstract; * denotes truncation. An English language filter was applied to this search. The key word search TI “classroom attainment” was piloted, but removed from the final search because it produced no results.

**Supplement 2: Stata syntax used in meta-analysis and meta-regression**

/* Fisher’s z-transformation of correlation coefficients (r) and sample sizes (n) */

generate z = atanh(r)

generate sez = sqrt(1/(n - 3))

/* Main meta-analysis */

metan z sez, random label(namevar = lead_author, yearvar = year)

/* Funnel plot */

metafunnel z sez

/* Egger test */

metabias z sez, egger

/* Meta-regression for follow-up period */

metareg z follow_up_period, wsse(sez)

/* Meta-regression for correlation coefficients broken down by age at baseline */

generate z_by_age = atanh(r_by_age)

generate sez_by_age = sqrt(1/(n_by_age - 3))

metareg z_by_age baseline_age, wsse(sez_by_age)

**Table S2: Full study characteristics table**

| Lead author, year | Country | Cohort name | n | Age and grade at depression measurement | % male | Additional inclusion/exclusion criteria reported at the participant level | Depression measurement | Outcome(s) | Follow-up period | Total risk of bias score (maximum=11) |
| --- | --- | --- | --- | --- | --- | --- | --- | --- | --- | --- |
| Birchwood,  2012 | United Kingdom | N/A | 324 | M=15.55; Range=15-16; Year 11 | 48% | N/A | HADS (continuous) | GCSE points score; GSCE score weighted for number of GCSE entries (continuous) | 1 school term | 5 |
| Chen,  2000 | China | N/A | 431 | M=11.58 (SD=1.0); 6^th^ grade | 54% | N/A | CDI (continuous) | Chinese and maths scores (continuous) | 2 years | 8 |
| Chen,  2013 | China | N/A | 1155 | M=9.33 (SD=0.67); 3^rd^ grade | 50% | N/A | CDI (continuous) | Chinese, maths and English scores (continuous) | 1 year | 7 |
| Chen,  2019 | China | N/A | 1430 | M=15.43; 7^th^ and 10^th^ grade | 47% | N/A | CDI (continuous) | Chinese, maths and English scores (continuous) | 1 year | 9 |
| Da Fonseca,  2009 | France | N/A | 353 | M=13.25 (SD=4.3); Range=11-16 | 50% | N/A | CDI (continuous) | Maths grade (continuous) | <1 school term | 4 |
| Davies,  2018 | United Kingdom | Avon Longitudinal Study of Parents and Children | 4398 | Range=12 | 48% | N/A | SMFQ (continuous and binary) | Achieving five or more A*-C grades at GCSE (binary); Achieving three or more A*-C grades at A Level (binary) | 4 and 6 years | 7 |
| Denault,  2009 | Canada | N/A | 362 | M=12.38 (SD=0.42); 7^th^-9^th^ grade | 42% | N/A | CDI (continuous) | Maths and French scores (continuous) | Between 1 and 3 years | 7 |
| Fu,  2018 | China | N/A | 336 | M=14.08 (SD=0.58); 7^th^-8^th^ grade | 55% | N/A | CDI (continuous) | Chinese, maths and English scores (continuous) | 1 year | 7 |
| Hood,  2017 | Australia | N/A | 244 | M=13.6 (SD=1.24); Range=11-17; Years 7-10 | 65% | N/A | DASS (continuous) | Average grade in 'all subjects' (continuous) | 1 semester | 5 |
| Ialongo,  2001 | United States of America | N/A | 625 | Range=9-10^a^; 4^th^ grade | 52% | N/A | MFQ-PSF (binary) | Overall GPA of C or worse (binary) | 1-2 years | 9 |
| Jonsson,  2010 | Sweden | N/A | 588 | M=16.44 (SD=0.63)^a^; Range=16-17; First year of upper secondary school | 22% | Exclusion: Participants not included in national registers at age 30 unless previous information on graduation from higher education available | BDI, CES-D for children, DICA-R-A (binary) | Final GPA in upper secondary school (continuous); Attained an upper secondary school diploma by age 20 (binary); Graduated from higher education by age 30 (binary); Graduated given that they entered higher education (binary) | 2-14 years | 10 |
| Kim,  2013 | United States of America | N/A | 379 | M=13.04 (SD=0.73); Range=12-15; 7^th^-8^th^ grade | 46% | Inclusion: Chinese American families, both parents foreign-born | CES-D (continuous) | GPA (excluding physical education courses) and California Standards Test scores in maths and English (continuous) | 4 years | 6 |
| Kim,  2015 | United States of America | N/A | 350 | M=13.03 (SD=0.73);  Range=12-15; 7^th^-8^th^ grade | 46% | Inclusion: Chinese American families | CES-D (continuous) | GPA (excluding physical education courses) (continuous) | 4 years | 6 |
| Kingery,  2011 | United States of America | N/A | 365 | M=11.17; 5^th^ grade | 48% | N/A | CDI (continuous) | English, science, social studies and maths grades (continuous) | 6 months | 5 |
| Lepore,  2013 | United States of America | N/A | 498 | M=12.8 (SD=0.44); 7^th^ grade | 44% | N/A | CDI (continuous) | Cumulative GPA across English, maths, social studies and science (continuous) | 6 months | 6 |
| Liu,  2018 | China | N/A | 945 | M=10.16 (SD=0.17); 4^th^-5^th^ grade | 51% | N/A | CDI (continuous) | Chinese, maths and English grades (continuous) | 1 and 2 years | 8 |
| Luthar,  1995 | United States of America | N/A | 138 | M=15.2 (SD=1.0); 9^th^ grade | 49% | N/A | CDI (continuous) | Mean grade across four academic courses (continuous) | 6 months | 7 |
| McLeod,  2012 | United States of America | National Longitudinal Study of Adolescent Health (Add Health) | 4701 | M=16.07 (SD=1.13);  9^th^-12^th^ grade | 46% | N/A | CES-D (binary) | Post-Wave I high school GPA (continuous) | 1-3 years^a^ | 8 |
| Morales,  2006 | United States of America | The Metropolitan Area Child Study | 2745 | Range=6-11; 1^st^-4^th^ grade | Not reported | N/A | CBCL-TRF (continuous) | Reading and maths scores from Iowa Test of Basic Skills (continuous) | 2 years | 5 |
| Nishina,  2005 | United States of America | N/A | 1526 | 6^th^ grade | 45% | Exclusion: Students absent for 20 or more school days during either fall or spring semester | CDI (continuous) | GPA across all classes (continuous) | 1 semester | 5 |
| Pate,  2017 | United States of America | National Longitudinal Study of Adolescent Health (Add Health) | 7276 | M=14.71 (SD=1.09); Range=13-16; 7^th^-12^th^ grade | 46% | N/A | CES-D (continuous) | Cumulative GPA across all waves (continuous) | 7 years | 9 |
| Riglin,  2013 | United Kingdom | N/A | 202 | M=11.25 (SD=0.44); Year 7 | 55% | N/A | SMFQ (continuous) | English, maths and science scores (continuous) | 2 school terms | 7 |
| Rockhill,  2009 | United States of America | N/A | 521 | M=12.0 (SD=0.41)^b^; 6^th^ grade | 52% | Inclusion: Participants recruited according to whether they met criteria for conduct problem symptoms only, depression symptoms only, both or neither. | MFQ (categorical) | Cumulative GPA (continuous) | 6 months | 6 |
| Rothon,  2009 | United Kingdom | Research with East London Adolescents: Community Health Survey (RELACHS) | 1636 | Range=13-14; Year 9 | 50% | N/A | SMFQ (continuous) | Achieving five or more A*-C grades at GCSE (binary) | 1-3 years | 9 |
| Rothon,  2011 | United Kingdom | Research with East London Adolescents: Community Health Survey (RELACHS) | 2499 | Range=13-14; Year 9 | 49% | N/A | SMFQ (binary) | Achieving five or more A*-C grades at GCSE (binary) | 2 years | 7 |
| Schwartz,  2005 | United States of America | N/A | 199 | M=9.02 (SD=0.57); Range=8.01-10.57; 3^rd^-4^th^ grade | 53% | Exclusion: children who were repeatedly absent, removed from class for administrative or disciplinary issues, or unable to participate due to involvement in specialised educational activities. | CDI (continuous) | GPA and scores on maths and reading subscales of the Stanford Achievement Test - Ninth Edition (continuous) | 1 year | 6 |
| Shahar,  2006 | United States of America | N/A | 460 | Range=11-14^b^; 6^th^-7^th^ grade | 49%^b^ | N/A | BDI (continuous) | GPA in English/reading, maths, social studies and science (continuous) | 1 year | 7 |
| Steele,  2000 | United States of America | Family Health Project (FHP) | 129 | M=8.78 (SD=1.69); Range=6-11 | 48% | Inclusion: Mothers self-identified as being HIV-negative, African American | CDI (continuous) | GPA across each academic subject (continuous) | 12-14 months | 8 |
| Wang,  2014 | United States of America | N/A | 935 | 10^th^ grade | 51% | N/A | CDI (continuous) | English, maths, science and social sciences grades (continuous) | 1 year minimum | 9 |
| Weidman,  2015 | United States of America | N/A | 130 | M=11.17 (SD=0.38);  6^th^-9^th^ grade | 60% | N/A | CDI (continuous) | GPA in English, maths, history/social studies, and science (continuous) | Up to 4 years | 8 |
| Zhang,  2019 | China | Longitudinal Study of Chinese Children | 648 | M=11.18 (SD=0.35); Range=95% of children aged 11.15 to 11.21^a^; 5^th^-8^th^ grade | 54% | N/A | CDI (continuous) | Chinese, maths and English scores (continuous) | 1 year | 9 |

Note: BDI=Beck Depression Inventory, CBCL-TRF=Child Behaviour Checklist - Teacher Report Form, CDI=Children's Depression Inventory, CES-D=Center for Epidemiologic Studies of Depression Scale, DASS=Depression Anxiety Stress Scale, DICA-R-A=Diagnostic Interview for Children and Adolescents in the revised form according to DSM-III-R for adolescents, GCSE=General Certificate of Secondary Education, GPA=Grade Point Average, HADS=Hospital Anxiety and Depression Scale, M=Mean, MFQ=Mood and Feelings Questionnaire, MFQ-PSF=Mood and Feelings Questionnaire - Parent Short Form, N/A=not applicable, SD=Standard Deviation, SMFQ=Short Mood and Feelings Questionnaire

a. Information provided or confirmed by the author

b. Estimated from another article which made use of the same or similar data, as recommended by the author

**Table S3: Risk of bias assessment scores**

| **Reference** | **Exposed cohort representative of target population (0-1)** | **Exposed and non-exposed groups drawn from the same cohort**  **(0-1)** | **Ascertainment of exposure using standardised diagnostic measure or a named measurement instrument**  **(0-1)** | **Adjustment made for baseline/prior educational attainment**  **(0-1)** | **Sample size justified and satisfactory**  **(0-1)** | **Controls for age and gender (0-1) and any other additional factor (0-1) either in selection of cohort or adjusted/stratified analysis** | **Outcome ascertained from school or administrative records**  **(0-1)** | **Follow-up ≥ 1 year**  **(0-1)** | **Follow-up complete, or unlikely to introduce bias (<20% lost or attrition described and accounted for in analysis)**  **(0-1)** | **Statistical test reporting clear, appropriate and complete**  **(0-1)** | **Total score**  **(0-11)** |
| --- | --- | --- | --- | --- | --- | --- | --- | --- | --- | --- | --- |
| Birchwood  2012 | 0 | 1 | 1 | 0 | 0 | 2 | 1 | 0 | 0 | 0 | 5 |
| Chen  2000 | 1 | 1 | 1 | 1 | 0 | 1 | 1 | 1 | 1 | 0 | 8 |
| Chen  2013 | 1 | 1 | 1 | 0 | 0 | 0 | 1 | 1 | 1 | 1 | 7 |
| Chen  2019 | 1 | 1 | 1 | 1 | 0 | 1 | 1 | 1 | 1 | 1 | 9 |
| Da Fonesca  2009 | 0 | 1 | 1 | 0 | 0 | 1 | 1 | 0 | 0 | 0 | 4 |
| Davies  2018 | 0 | 1 | 1 | 0 | 0 | 2 | 1 | 1 | 0 | 1 | 7 |
| Denault  2009 | 1 | 1 | 1 | 0 | 0 | 0 | 1 | 1 | 1 | 1 | 7 |
| Fu  2018 | 1 | 1 | 1 | 0 | 0 | 0 | 1 | 1 | 1 | 1 | 7 |
| Hood  2017 | 0 | 1 | 1 | 0 | 0 | 0 | 1 | 0 | 1 | 1 | 5 |
| Ialongo  2001 | 1 | 1 | 1 | 1 | 0 | 2 | 1 | 1 | 1 | 0 | 9 |
| Jonsson  2010 | 1 | 1 | 1 | 1 | 0 | 2 | 1 | 1 | 1 | 1 | 10 |
| Kim  2013 | 0 | 1 | 1 | 0 | 0 | 1 | 1 | 1 | 0 | 1 | 6 |
| Kim  2015 | 0 | 1 | 1 | 0 | 0 | 1 | 1 | 1 | 0 | 1 | 6 |
| Kingery  2011 | 0 | 1 | 1 | 0 | 0 | 0 | 1 | 0 | 1 | 1 | 5 |
| Lepore  2013 | 0 | 1 | 1 | 0 | 0 | 2 | 1 | 0 | 1 | 0 | 6 |
| Liu  2018 | 1 | 1 | 1 | 0 | 0 | 2 | 1 | 1 | 1 | 0 | 8 |
| Luthar  1995 | 0 | 1 | 1 | 1 | 0 | 2 | 1 | 0 | 1 | 0 | 7 |
| McLeod  2012 | 1 | 1 | 1 | 0 | 0 | 2 | 1 | 1 | 0 | 1 | 8 |
| Morales  2006 | 0 | 1 | 1 | 0 | 0 | 0 | 1 | 1 | 0 | 1 | 5 |
| Nishina  2005 | 0 | 1 | 1 | 0 | 0 | 1 | 1 | 0 | 0 | 1 | 5 |
| Pate  2017 | 1 | 1 | 1 | 0 | 0 | 2 | 1 | 1 | 1 | 1 | 9 |
| Riglin  2013 | 0 | 1 | 1 | 1 | 0 | 2 | 1 | 0 | 1 | 0 | 7 |
| Rockhill  2009 | 0 | 1 | 1 | 0 | 0 | 2 | 1 | 0 | 0 | 1 | 6 |
| Rothon  2009 | 0 | 1 | 1 | 1 | 0 | 2 | 1 | 1 | 1 | 1 | 9 |
| Rothon  2011 | 1 | 1 | 1 | 0 | 0 | 2 | 1 | 1 | 0 | 0 | 7 |
| Schwartz  2005 | 0 | 1 | 1 | 0 | 0 | 1 | 1 | 1 | 1 | 0 | 6 |
| Shahar  2006 | 0 | 1 | 1 | 1 | 0 | 1 | 1 | 1 | 1 | 0 | 7 |
| Steele  2000 | 1 | 1 | 1 | 1 | 0 | 1 | 1 | 1 | 1 | 0 | 8 |
| Wang  2014 | 1 | 1 | 1 | 1 | 0 | 2 | 1 | 1 | 1 | 0 | 9 |
| Weidman  2015 | 0 | 1 | 1 | 1 | 0 | 2 | 1 | 1 | 1 | 0 | 8 |
| Zhang  2019 | 1 | 1 | 1 | 1 | 0 | 2 | 1 | 1 | 0 | 1 | 9 |

**Table S4: Data used in meta-analysis and in meta-regression of follow-up period**

| **lead_author** | **year** | **follow_up_period** | **r** | **n** |
| --- | --- | --- | --- | --- |
| Birchwood | 2012 | 3 | -0.29 | 324 |
| Chen | 2000 | 24 | -0.25 | 431 |
| Chen | 2013 | 12 | -0.23 | 1171 |
| Chen | 2019 | 12 | -0.14 | 1430 |
| Da Fonseca | 2009 | 3 | -0.14 | 353 |
| Denault | 2009 | 24 | -0.17 | 362 |
| Fu | 2018 | 12 | -0.22 | 336 |
| Hood | 2017 | 3 | -0.08 | 244 |
| Kim | 2013 | 48 | -0.16 | 379 |
| Kim | 2015 | 48 | -0.14 | 350 |
| Kingery | 2011 | 6 | -0.23 | 365 |
| Lepore | 2013 | 6 | -0.14 | 485 |
| Liu | 2018 | 18 | -0.25 | 945 |
| Luthar | 1995 | 6 | -0.04 | 138 |
| Morales | 2006 | 24 | -0.11 | 2745 |
| Nishina | 2005 | 3 | -0.12 | 758 |
| Pate | 2017 | 84 | -0.20 | 5931 |
| Riglin | 2013 | 6 | -0.21 | 202 |
| Shahar | 2006 | 12 | -0.27 | 406 |
| Steele | 2000 | 13 | -0.19 | 129 |
| Weidman | 2015 | 24 | -0.32 | 129 |
| Zhang | 2019 | 12 | -0.26 | 642 |

**Table S5: Data used in meta-regression of age at baseline**

| **lead_author** | **year** | **baseline_age** | **r_by_age** | **n_by_age** |
| --- | --- | --- | --- | --- |
| Birchwood | 2012 | 15.55 | -0.29 | 324 |
| Chen | 2000 | 11.58 | -0.25 | 431 |
| Chen | 2013 | 9.33 | -0.23 | 1171 |
| Chen | 2019 | 15.43 | -0.14 | 1430 |
| Da Fonseca | 2009 | 13.25 | -0.14 | 353 |
| Denault | 2009 | 12.5 | -0.17 | 362 |
| Denault | 2009 | 13.5 | -0.13 | 362 |
| Denault | 2009 | 14.5 | -0.27 | 362 |
| Fu | 2018 | 14.08 | -0.22 | 336 |
| Hood | 2017 | 13.6 | -0.08 | 244 |
| Kim | 2013 | 13.04 | -0.16 | 379 |
| Kim | 2015 | 13.03 | -0.14 | 350 |
| Kingery | 2011 | 11.17 | -0.23 | 365 |
| Lepore | 2013 | 12.8 | -0.14 | 485 |
| Liu | 2018 | 10.16 | -0.25 | 945 |
| Liu | 2018 | 11.16 | -0.25 | 945 |
| Luthar | 1995 | 15.2 | -0.04 | 138 |
| Morales | 2006 | 8.5 | -0.11 | 2745 |
| Nishina | 2005 | 11.5 | -0.12 | 758 |
| Pate | 2017 | 14.71 | -0.20 | 5931 |
| Riglin | 2013 | 11.25 | -0.21 | 202 |
| Shahar | 2006 | 12.5 | -0.27 | 406 |
| Steele | 2000 | 8.78 | -0.19 | 129 |
| Weidman | 2015 | 11.17 | -0.41 | 129 |
| Weidman | 2015 | 12.5 | -0.22 | 129 |
| Weidman | 2015 | 13.5 | -0.31 | 129 |
| Weidman | 2015 | 14.5 | -0.32 | 129 |
| Zhang | 2019 | 11.18 | -0.29 | 645 |
| Zhang | 2019 | 12.18 | -0.21 | 640 |
| Zhang | 2019 | 13.18 | -0.26 | 639 |
| Zhang | 2019 | 14.18 | -0.29 | 639 |

**Supplement 3: Formula back-transforming Fisher’s *z* to a correlation coefficient**

$$r=\frac{e^{(2x0.19)}-1}{e^{(2x0.19)}+1}=0.19$$

**Figure S1: Funnel plot**

**
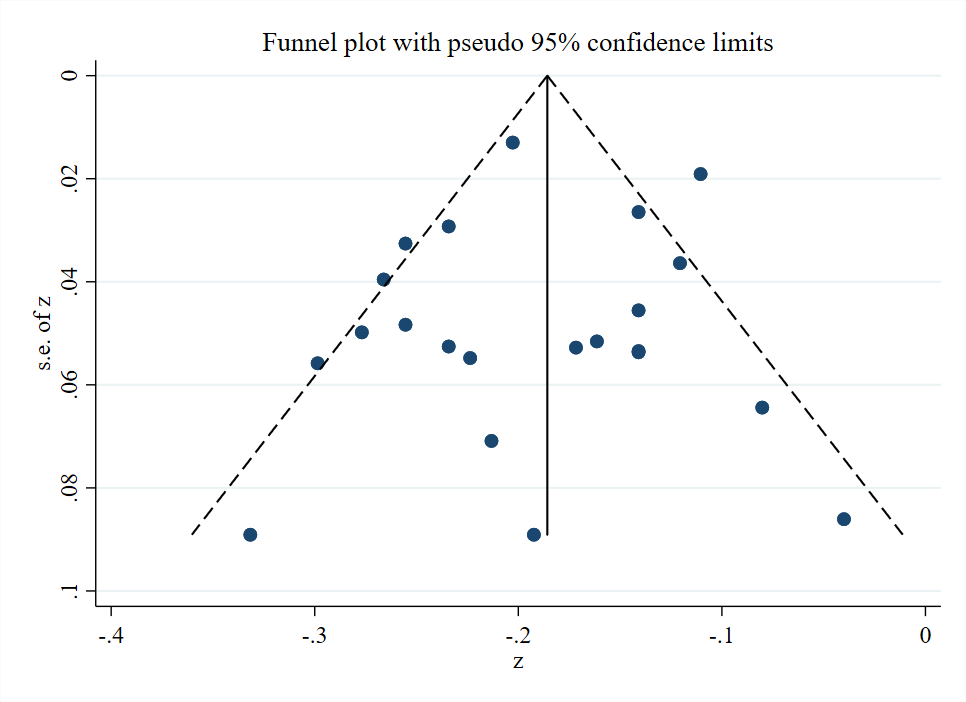
**

**Table S6: Results from stratified meta-analyses**

| **Stratified analyses** | **Pooled Fisher’s *z* (95% CI)** | **Cochran’s Q** | **I^2^** |
| --- | --- | --- | --- |
| Risk of bias |  |  |  |
| *High (n=6)* | -0.16 (-0.22 to -0.10) | Q(5)=14.70, p=0.012 | 66.0% |
| *Low (n=16)* | -0.21 (-0.23 to -0.18) | Q(15)=25.18, p=0.048 | 40.4% |
| Depression measurement instrument |  |  |  |
| *CDI (n=14)* | -0.20 (-0.23 to -0.16) | Q(13)=27.43, p=0.011 | 52.6% |
| *CES-D (n=3)* | -0.20 (-0.22 to -0.17) | Q(2)=1.76, p=0.414 | 0.0% |
| Country of study |  |  |  |
| *USA (n=11)* | -0.17 (-0.21 to -0.13) | Q(10)=30.26, p=0.001 | 66.9% |
| *China (n=6)* | -0.23 (-0.27 to -0.18) | Q(5)=12.18, p=0.032 | 58.9% |
| *UK (n=2)* | -0.27 (-0.35 to -0.18) | Q(1)=0.90, p=0.344 | 0.0% |

**Table S7: Results of multivariable analyses (only results where all confounders have been adjusted for are captured)**

| Lead author, year | Depression measurement | Attainment outcome | Confounders adjusted for | Any significant results following full adjustment | Results following adjustment | Effect modifiers investigated | Mediators investigated |
| --- | --- | --- | --- | --- | --- | --- | --- |
| Birchwood,  2012 | HADS  (continuous) | GCSE points score  (continuous) | Cross-school differences, cognitive ability, motivation, anxiety, aggression, lifetime attention deficit hyperactivity disorder | Yes | Beta=-0.078, t=-1.872, p=0.062 | None | None |
|  |  | GSCE score weighted for number of GCSE entries  (continuous) |  |  | Beta=-0.102, t=-2.446, p=0.015 |  |  |
| Chen,  2000 | CDI  (continuous) | Chinese and maths scores  (continuous) | Time 1 academic achievement, gender | No | Beta=-0.06, R^2^ change=0.01, F change=2.43, p>0.05 | Gender | None |
| Chen,  2019 | CDI  (continuous) | Chinese, maths and English scores  (continuous) | Gender, Time 1 academic achievement, Time 1 group-level depression | Yes | b=-0.22, SE=0.09, t=-2.54, p<0.05, 95% CI=-0.39 to -0.05, rES=0.07 | School year | None |
| Da Fonseca,  2009 | CDI  (continuous) | Maths grade  (continuous) | Entity theory, maths ability | Yes | Beta=-0.26, F(1,349)=-6.03, p<0.001 | None | None |
| Davies,  2018 | SMFQ  (binary) | Not achieving five or more A*-C grades at GCSE (binary) | Gender, social class, housing, maternal education, IQ at age 8, total SDQ score at age 8, psychotic experiences at age 12 | Yes | OR=1.63, 95% CI=1.06 to 2.50, p=0.026 | None | None |
|  |  | Not achieving three or more A*-C grades at A Level (binary) |  |  | OR=1.03, 95% CI=0.69 to 1.54, p=0.880 |  |  |
|  | SMFQ  (continuous) | Not achieving five or more A*-C grades at GCSE (binary) |  |  | OR=1.01, 95% CI=0.99 to 1.04, p=0.356 |  |  |
|  |  | Not achieving three or more A*-C grades at A Level (binary) |  |  | OR=0.97, 95% CI=0.95 to 0.99, p=0.014 |  |  |
| Ialongo,  2001 | MFQ-PSF  (binary) | Overall GPA of C or worse  (binary) | Ethnicity, free lunch status, intervention condition, parent education, family structure, first-grade standardized achievement | Yes | Boys: OR=10.31, 95% CI=1.97-54.10 | Gender | None |
|  |  |  |  |  | Girls: Not significant (not reported in full) |  |  |
|  |  |  | Ethnicity, free lunch status, intervention condition, parent education, family structure, child self-reports of depression at first grade |  | Boys: OR=9.14, 95% CI=1.69 to 49.36 |  |  |
|  |  |  |  |  | Girls: OR=0.84, 95% CI=0.34 to 2.05 |  |  |
| Jonsson,  2010 | BDI, CES-D for children, DICA-R-A (binary) | Graduation from higher education by age 30 (binary) | Socioeconomic status, maternal education, GPA in compulsory school | Yes | Boys: OR=0.27, 95% CI=0.08 to 0.93, p<0.05 | Gender | None |
|  |  |  |  |  | Girls: OR=0.93, 95% CI=0.58 to 1.49, p>0.05 |  |  |
|  | DICA-R-A  (binary) (only adolescents with major depression included) |  |  |  | Boys: OR=0.16, 95% CI=0.03 to 0.94 |  |  |
|  |  |  |  |  | Girls: OR=0.82, 95% CI=0.50 to 1.36 |  |  |
| Lepore,  2013 | CDI  (continuous) | Cumulative GPA across English, maths, social studies and science (continuous) | Final path model included sleep problems, absence rate, intrusive thoughts, and community violence. | No | Beta=0.06, p>0.05 | Gender, ethnicity | None |
|  |  |  | Final path model included sleep problems, absence rate, intrusive thoughts, and peer victimisation. |  | Beta=0.08, p>0.05 |  |  |
| Liu,  2018 | CDI  (continuous) | Chinese, maths and English grades  (continuous) | Cross-lagged models controlled for concurrent associations including victimisation | Yes | Grade 4 to 5 (direct): b=-0.09, p<0.01 | Gender | Victimisation |
|  |  |  |  |  | Grade 5 to 6 (direct): b=-0.07, p<0.01 |  |  |
|  |  |  |  |  | Grade 4 to 6 (indirect): b=-0.06, SE=0.01, 95% CI=-0.08 to -0.03, p<0.01 |  |  |
| Luthar,  1995 | CDI  (continuous) | Mean grade across four academic courses  (continuous) | Gender, age, Time 1 grades, assertiveness, sociability, leadership, anxiety, internalising symptoms, externalising symptoms (adjustments also made for ethnicity, but results for this association not fully reported) | No | Beta=-0.03, R^2^=0.00, p>0.05 | Gender | None |
| McLeod,  2012 | CES-D  (binary) | Post-Wave I high school GPA  (continuous) | Wave I vocabulary test scores, learning disability or special education services in the year prior to wave I, gender, race, whether the youth's family received public assistance, highest level of parental education, family income, family structure, grade level, age, attention problems, delinquency, cigarette use, alcohol use, drug use | No | b=-0.05, SE=0.05, p>0.05 | Gender | None |
| Pate,  2017 | CES-D  (continuous) | Cumulative GPA across all waves  (continuous) | School connectedness, age, socioeconomic status, biological sex, race | Yes | Multiply imputed: Beta=-0.27, SE=0.04, p<0.001 | School connectedness | School connectedness |
|  |  |  |  |  | Not imputed: Beta=-0.28, SE=0.03, p<0.001 |  |  |
| Riglin,  2013 | SMFQ  (continuous) | English, maths and science scores  (continuous) | Conduct problems, across time stability in academic attainment | No | Beta=-0.06, p>0.05 | Gender, school | None |
| Rockhill,  2009 | MFQ  (categorical) | Cumulative GPA  (continuous) | Age, sex, family income, race | Yes | Unstandardised beta=-0.24, SE=0.10, standardised beta=-0.091, SE=0.10, t=-2.45, p<0.05 | None | Social competence and social support |
| Rothon,  2009 | SMFQ  (continuous) | Achieving five or more A*-C grades at GCSE  (binary) | Cohort, eligibility for free school meals, ethnicity, achievement at ages 13-14 | No | Not fully reported, but non-significant | Gender, free school meals, ethnicity | None |
| Rothon,  2011 | SMFQ  (binary) | Achieving five or more A*-C grades at GCSE  (binary) | Gender, ethnicity, eligibility for free school meals, psychological distress, self-esteem, parents help with problems, parents encourage to do well, aspiration to remain in education beyond the age of 16 | No | OR=1.09, 95% CI=0.80 to 1.47 | None | None |
|  |  |  | Gender, ethnicity, eligibility for free school meals, psychological distress, self-esteem, parents help with problems, parents encourage to do well, aspiration to do A levels |  | OR=1.08, 95% CI=0.81 to 1.44 |  |  |
| Schwartz,  2005 | CDI  (continuous) | GPA and scores on maths and reading subscales of the Stanford Achievement Test - Ninth Edition  (continuous) | Final path model included peer victimisation at times 1 and 2, depression at time 2 | Yes | Beta=-0.21, p<0.05 | None | None |
| Shahar,  2006 | BDI  (continuous) | GPA in English/reading, maths, social studies and science  (continuous) | Prior GPA, gender, self-criticism | Yes | Beta=-0.12, p<0.01 | Gender, self-criticism | None |
| Steele,  2000 | CDI  (continuous) | GPA across each academic subject  (continuous) | Gender, final grades at Time 1 | Yes | B=0.03, SE=0.01, Beta=-0.17, p<0.01 | Gender | None |
| Wang,  2014 | CDI  (continuous) | English, maths, science and social sciences grades  (continuous) | School involvement, home involvement, academic socialisation, GPA at 10th grade, problem behaviour, gender, socioeconomic status, ethnicity | Yes | Beta=-0.10, p<0.01 | None | None |
| Weidman,  2015 | CDI  (continuous) | GPA in English, maths, history/social studies, and science  (continuous) | Depression and GPA intercept and slope, negative affect | Yes | Grade 7: b=-0.20, p>0.10 | None | None |
|  |  |  |  |  | Grade 8: b=-0.28, p<0.10 |  |  |
|  |  |  |  |  | Grade 9: b=-0.19, p>0.10 |  |  |
|  |  |  |  |  | Grade 10: b=-0.59, p<0.10 |  |  |
|  |  |  | Depression and GPA intercept and slope, positive affect |  | Grade 7: b=-0.35, p>0.10 |  |  |
|  |  |  |  |  | Grade 8: b=-0.26, p<0.10 |  |  |
|  |  |  |  |  | Grade 9: b=-0.44, p<0.05 |  |  |
|  |  |  |  |  | Grade 10: b=-0.89, p<0.05 |  |  |
|  |  |  | Depression and GPA intercept and slope, either IQ or gender |  | *bs*=-0.19 to -0.85 |  |  |
| Zhang,  2019 | CDI  (continuous) | Chinese, maths and English scores  (continuous) | Socioeconomic status, negative maternal parenting, attention problems, externalising problems | Yes | Grade 5 to 6: Beta=-0.13, SE=0.04, p<0.001 | Gender | None |
|  |  |  |  |  | Grade 6 to 7: Beta=-0.07, SE=0.03, p<0.05 |  |  |
|  |  |  |  |  | Grade 7 to 8: Beta=0.00, SE=0.02, p>0.05 |  |  |
|  |  |  |  |  | Grade 8 to 9: Beta=-0.03, SE=0.02, p>0.05 |  |  |

Note: BDI=Beck Depression Inventory, CDI=Children's Depression Inventory, CES-D=Center for Epidemiologic Studies of Depression Scale, DICA-R-A=Diagnostic Interview for Children and Adolescents in the revised form according to DSM-III-R for adolescents, GCSE=General Certificate of Secondary Education, GPA=Grade Point Average, HADS=Hospital Anxiety and Depression Scale, MFQ=Mood and Feelings Questionnaire, MFQ-PSF=Mood and Feelings Questionnaire - Parent Short Form, OR=Odds Ratio, SDQ=Strength and Difficulties Questionnaire, SMFQ=Short Mood and Feelings Questionnaire
